# Effects of admission hyperglycemia and intravenous thrombolysis allocation in acute basilar artery occlusion after endovascular treatment: Analysis of the ATTENTION registry

**DOI:** 10.1016/j.neurot.2023.10.013

**Published:** 2023-12-19

**Authors:** Rui Li, Thanh N. Nguyen, Pengfei Xu, Chunrong Tao, Wenhuo Chen, Zhihua Cao, Yamei Yin, Li Wang, Juan Chen, Zi Wang, Jun Sun, Lang Chen, Peng Hao, Shuo Feng, Xinfeng Liu, Wei Hu, the ATTENTION (Endovascular Treatment for Acute Basilar Artery Occlusion) Trial Investigators

**Affiliations:** a Department of Neurology, The First Affiliated Hospital of USTC, Division of Life Sciences and Medicine, University of Science and Technology of China, Hefei, China; b Department of Neurology, Boston Medical Center, Boston University Chobanian & Avedisian School of Medicine, Boston, MA, USA; c Department of Neurology, Zhangzhou Affiliated Hospital of Fujian Medical University, Zhangzhou, China; d Department of Neurology, Xiangyang No.1 People's Hospital, Hubei University of Medicine, Xiangyang, China; e Department of Neurology, Provincial Hospital Affiliated to Anhui Medical University, Hefei, China

**Keywords:** Basilar artery occlusion, Endovascular treatment, Hyperglycemia, Intravenous thrombolysis, Ischemic stroke

## Abstract

This study was to investigate the admission hyperglycemia and modified effect of intravenous thrombolysis (IVT) on clinical outcomes in acute basilar artery occlusion (BAO) patients receiving endovascular treatment (EVT). We prospectively recruited acute BAO patients from 48 stroke centers across 22 Chinese provinces in the ATTENTION registry from 2017 to 2021. Hyperglycemia on admission was defined as glucose ≥7.8 ​mmol/L. We performed multivariable logistic regression analysis to evaluate the correlation of hyperglycemia on admission with the primary outcome defined as a modified Rankin scale (mRS) score of <4 ​at 90 days, and the secondary outcomes defined as successful recanalization, mRS 0–1 and 0–2 ​at 90 days. Safety outcomes were symptomatic intracranial hemorrhage (sICH) and mortality within 90 days. There were 1195 patients with acute BAO treated with EVT of whom 519 had hyperglycemia on admission. Hyperglycemia on admission was inversely associated with favorable neurological outcomes (mRS 0–3: adjusted odd ratio [aOR] 0.69, 95 ​% confidence intervals [CI] 0.54–0.89, P ​= ​0.004; mRS 0–1: aOR 0.67, 95 ​% CI 0.50–0.90, P ​= ​0.008; mRS 0–2: aOR 0.73, 95 ​% CI 0.56–0.95; P ​= ​0.02). Hyperglycemia on admission was not correlated to sICH nor successful recanalization. In the subgroup of BAO patients treated with direct EVT, those with hyperglycemia on admission had a higher mortality rate, and overall worse clinical outcomes at 90 days than patients without hyperglycemia. A significant interaction was observed between IVT and hyperglycemia on admission (P_interaction_ ​= ​0.017). In patients with acute BAO treated with EVT, hyperglycemia on admission was associated with worse functional outcomes at 90 days but was not correlated with sICH nor successful recanalization. The effect of admission hyperglycemia appears to be modified by IVT allocation. Unique identifier: ChiCTR2000041117.

## Introduction

Acute basilar artery occlusion (BAO) is a devastating medical condition with a high morbidity and mortality rate [1]. The benefit of endovascular treatment (EVT) on patients with acute BAO has been confirmed in prospective registries and recent randomized clinical trials [[2], [3], [4], [5]]. The BAOCHE (the Basilar Artery Occlusion Chinese Endovascular trial) and ATTENTION (Endovascular treatment for acute basilar artery occlusion) demonstrated that EVT correlated with better functional outcomes compared to patients who were medically managed [4,5]. While EVT is considered as an effective treatment for acute BAO, more than half of patients are left severely disabled [6]. Therefore, early detection of patients who would benefit from EVT could enable clinicians to choose the best clinical strategy for each individual.

Hyperglycemia is a known risk factor for acute ischemic stroke (AIS), and may be associated with infarct volume expansion, secondary hemorrhage, high mortality, and poor outcome [7,8]. The mechanism by which hyperglycemia may augment ischemic injury may be mediated by endothelial dysfunction, oxidative stress, and impaired fibrinolysis [9]. Several studies reported that large vessel occlusion (LVO) patients with hyperglycemia on admission who undergo EVT have a higher mortality rate and worse clinical outcomes than patients without hyperglycemia [[10], [11], [12]]. A recent meta-analysis performed on AIS patients who had mechanical thrombectomy (MT) showed that higher glucose levels on admission were associated with worse long-term outcome [13]. One study [14] suggested that baseline serum glucose levels can be used as a modifiable parameter to increase the likelihood of improving functional outcome in BAO. Yet the findings of this study were limited by their sample size, and further analyses on larger cohorts of patients are needed. It remains uncertain whether hyperglycemia on admission has an effect on the clinical outcomes of acute BAO patients treated with EVT. Moreover, the modified effect of intravenous thrombolysis (IVT) allocation was also unknown. In this study, we aimed to investigate those effects in patients with acute BAO receiving EVT.

## Methods

### Study population

We utilized data from the ATTENTION prospective registry [3], a nationwide registry comprising consecutive patients with BAO treated with EVT versus medical management from 48 stroke centers in China between March 2017 and February 2021. This study was approved by the ethics committee of the First Affiliated Hospital of University of Science and Technology of China (2020KY-202). All patients, or their legal representatives, signed informed consent prior to enrollment in the ATTENTION registry (https://www.chictr.org.cn; ChiCTR2000041117). Detailed study methods and patient eligibility criteria have been reported previously [3].

### Treatments

EVT includes aspiration, stent retriever, balloon angioplasty, intra-arterial thrombolysis, intracranial stent deployment or any combination of these methods at the discretion of the treating physician. IVT was given in the first 4.5 ​h from stroke onset with a standard dosage of alteplase (0.9 ​mg/kg in 1 ​h, maximum 90 ​mg) [15]. Bridging therapy was defined as IVT followed by EVT (IVT ​+ ​EVT).

### Data collection

We collected detailed patient information including demographic parameters, clinical findings including the National Institute of Health Stroke Scale (NIHSS), laboratory data, imaging findings, type of treatment received, complications and functional outcomes at 90 days. The posterior circulation-Alberta Stroke Program Early Computed Tomography Score (PC-ASPECTS) was used to determine early ischemic changes. Hyperglycemia on admission was defined as serum glucose on admission ≥7.8 ​mmol/L [10,16].

BAO was divided into proximal, middle and distal BAO from the vertebrobasilar junction to the origin of the posterior cerebral artery [17]. In addition, we recorded the estimated time of BAO to admission (OTA).

### Outcomes

The primary outcome was a favorable functional outcome defined as a modified Rankin Scale (mRS) score of 0–3 ​at 90 (±14) days. Secondary outcomes were the scores of mRS 0–1, mRS 0–2 ​at 90 (±14) days and successful recanalization (modified Thrombolysis in Cerebral Infarction [mTICI] score 2b-3) [18]. Safety outcomes included all-cause mortality within 90 days and symptomatic intracranial hemorrhage (sICH) within 3 days after EVT. sICH was identified as any intracranial hemorrhage on brain imaging with a ≥4 point increase of the NIHSS score from baseline [19].

### Statistical analysis

Continuous data were expressed as mean ​± ​standard deviation (SD) for normal data or median and interquartile range (IQR) for skewed data. Mann-Whitney *U* test was used to analyze skewed data, and student's t-test for normal data. Categorical data were expressed as numbers with their frequency (%) and compared with the Pearson χ2 test or Fisher's exact test.

We performed multivariable logistic regression analysis, adjusting for potential confounding factors, to evaluate the correlation of hyperglycemia on admission with the primary outcome, secondary outcomes, and safety outcomes of EVT in acute BAO patients. We obtained crude and adjusted odds ratios (aORs) with 95 ​% confidence intervals (CIs). Subgroups were divided based on age (≤74 years vs. >74 years), gender (female vs. male), baseline NIHSS score (≤10 vs. >10), OTA (≤6 ​h vs. >6 ​h), baseline PC-ASPECTS (≤8 vs. >8), level of occlusion sites (proximal BAO, middle BAO and distal BAO), coronary heart disease, hyperlipidemia, hypertension, smoking, atrial fibrillation, IVT, diabetes mellitus (DM), stroke or transient ischemic attack (TIA). The multiplicative term was calculated for subgroup analysis, and a P for interaction <0.05 was regarded as statistically heterogeneous. In addition, we applied binary logistic regression analysis to evaluate the correlation of hyperglycemia on admission with the primary outcome, secondary outcomes, and safety outcomes in different subgroups (Direct EVT and Bridging therapy). Moreover, subgroups in different window time (OTA ≤4.5 ​h and >4.5 ​h) were analyzed. The primary outcome of admission hyperglycemia in patients with DM and those without DM was also evaluated with logistic regression.

In the ordinal logistic regression analysis, we analyzed the adjusted common odds ratio (OR) as a shift towards a worse outcome of mRS scores. We further evaluated the pattern and magnitude of associations of variables with primary outcome and mortality at 90 days using a logistic regression model with restricted cubic splines (RCS) with 4 knots at the 5th, 35th, 65th and 95th centiles, adjusting for covariates. The probability of clinical outcomes according to glucose levels on admission was plotted separately for patients with IVT allocation.

The predictive ability of glucose levels for clinical outcomes was defined by the area under the curve (AUC) in receiver operating characteristic (ROC) analyses. The optimal cut-off value was based on the greatest value of the Youden Index for identifying sensitivity and specificity.

To avoid case deletion in missing data in univariate and multivariable analyses, we performed multiple imputation with five datasets to impute the missing data by chained equations (MICE). The imputation procedure was performed under the missing-at-random assumption. The imputation model included all variables in the analysis model to compute the missing values. We combined the estimates obtained from the different imputed datasets using Rubin rules. Statistical analyses were conducted with SPSS 26.0 (Chicago, IL, USA). Figures were drawn using Microsoft Excel 2019 software and STATA version 15 (College Station, Texas, USA). P ​< ​0.05 was regarded as statistical significance.

## Results

### Baseline characteristics

**Fig. 1:**
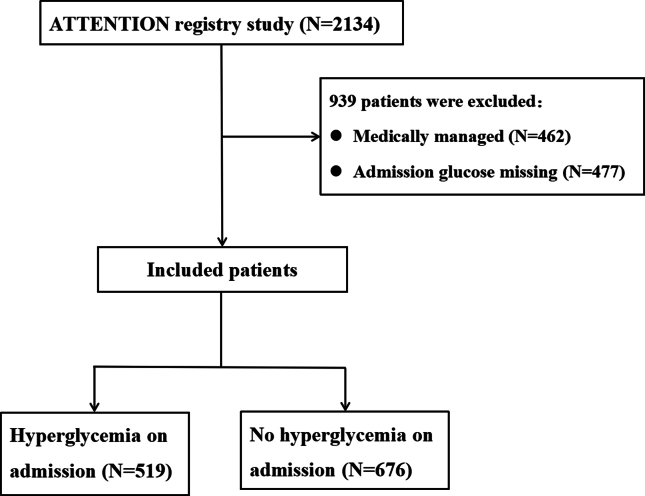
**Flowchart of patient selection.** ATTENTION: Endovascular Treatment for Acute Basilar Artery Occlusion.

A total of 2134 patients with acute BAO were enrolled in the ATTENTION registry. 462 patients were excluded because they were medically managed; 477 patients for whom admission glucose was missing (Fig. 1). 1195 acute BAO patients treated with EVT were included in the final study (519 patients with hyperglycemia and 676 without hyperglycemia on admission). The baseline characteristics of patients with hyperglycemia versus without hyperglycemia on admission are displayed in Table 1. Compared with non-hyperglycemic patients, those with hyperglycemia on admission were less likely to be male (341 [65.7 ​%] vs. 496 [73.4 ​%]; P ​= ​0.004), had a higher rate of hypertension (386 [74.4 ​%] vs. 436 [64.5 ​%]; P ​< ​0.001) and DM (210 [40.5 ​%] vs. 84 [12.4 ​%]; P ​< ​0.001), had a lower frequency of being smokers (139 [26.8 ​%] vs. 232 [34.3 ​%]; P ​= ​0.005), had a higher median [interquartile range, IQR] systolic blood pressure (150 [134,166] vs. 145 [131,162]; P ​= ​0.005). We found no differences in other baseline variables between patients with and without hyperglycemia on admission.

**Table 1:** Baseline characteristics of patients with acute BAO after EVT with hyperglycemia versus without hyperglycemia on admission.

	Admission Hyperglycemia	Without Admission Hyperglycemia	P value
**Total**	519	676	
**Age, median (IQR), years**	66 (56, 73)	65 (56, 74)	0.665
**Male, no. (%)**	341 (65.7)	496 (73.4)	0.004
**Medical history, no. (%)**			
Hypertension	386 (74.4)	436 (64.5)	<0.001
Diabetes mellitus	210 (40.5)	84 (12.4)	<0.001
Hyperlipidemia	175 (33.7)	223 (33.0)	0.791
Coronary heart disease	82 (15.8)	88 (13.0)	0.172
History of stroke or TIA	126 (24.3)	161 (23.8)	0.853
AF	157 (30.3)	221 (32.7)	0.368
**Smoking, no. (%)**	139 (26.8)	232 (34.3)	0.005
**Blood pressure on admission, mm Hg**			
Systolic blood pressure, median (IQR)	150 (134, 166)	145 (131, 162)	0.005
Diastolic blood pressure, median (IQR)	85 (76, 96)	84 (75, 94)	0.205
**Baseline NIHSS score, median (IQR)**	21 (13, 29)	20 (12, 30)	0.763
**OTA, median (IQR), min**	342 (215, 595)	321 (221, 581)	0.777
**Location of occlusion, no. (%)**			
Proximal basilar artery	166 (32.0)	221 (32.7)	0.513
Middle basilar artery	177 (34.1)	210 (31.1)	
Distal basilar artery	176 (33.9)	245 (36.2)	
**Baseline PC-ASPECTS (≥8), no. (%)**	440 (84.8)	595 (88.0)	0.103
**IVT, no. (%)**	115 (22.2)	183 (27.1)	0.052

BAO: basilar artery occlusion; EVT: endovascular treatment; IQR: interquartile range; TIA: transient ischemic attack; AF: atrial fibrillation; NIHSS: National Institutes of Health Stroke Scale; OTA: estimated time of basilar artery occlusion to admission; PC-ASPECTS: posterior circulation Alberta Stroke Program Early Computed Tomography Score; IVT: intravenous thrombolysis; mRS: modified Rankin Scale.

### Clinical outcomes among acute BAO patients treated with EVT

The primary outcome, secondary outcomes, and safety outcomes for hyperglycemia on admission in acute BAO patients treated with EVT are shown in Table 2. After adjusting for potential confounder variables (age, sex, smoking, hypertension, hyperlipidemia, atrial fibrillation, baseline NIHSS score, IVT, location of occlusion, OTA, history of stroke or TIA, baseline PC-ASPECTS, and coronary heart disease), we observed that hyperglycemia on admission was associated with a decreased likelihood of achieving good functional outcomes (mRS score 0–3: aOR 0.69, 95 ​% CI 0.54–0.89, P ​= ​0.004; mRS score 0–1: aOR 0.67, 95 ​% CI 0.50–0.90, P = 0.008; mRS score 0–2: aOR 0.73, 95 ​% CI 0.56–0.95, P ​= ​0.020). We did not identify any difference in safety outcomes and successful recanalization between patients with or without hyperglycemia on admission although there was a trend for higher 90-day mortality in the hyperglycemia group (aOR 1.27, 95 ​% CI 0.98–1.64, P ​= ​0.068).

**Table 2:** Primary outcome, secondary outcomes, and safety outcomes for hyperglycemia in acute BAO patients treated with EVT.

	Crude OR (95 ​% CI)	P value	Adjusted OR (95 ​% CI)	P value
**Primary outcome**				
mRS 0–3 ​at 90 ​d	0.68 (0.54–0.87)	0.002	0.69 (0.54–0.89)	0.004
**Secondary outcomes**				
mRS 0–1 ​at 90 ​d	0.66 (0.50–0.88)	0.004	0.67 (0.50–0.90)	0.008
mRS 0–2 ​at 90 ​d	0.72 (0.56–0.92)	0.009	0.73 (0.56–0.95)	0.020
Successful recanalization	0.84 (0.60–1.17)	0.299	0.90 (0.64–1.26)	0.538
**Safety outcomes**				
sICH at 3 ​d	1.19 (0.71–2.00)	0.507	1.11 (0.66–1.89)	0.687
Mortality at 90 ​d	1.32 (1.04–1.67)	0.023	1.27 (0.98–1.64)	0.068

BAO: basilar artery occlusion; EVT: endovascular treatment; mRS: modified Rankin Scale; sICH: symptomatic intracranial hemorrhage; AF: atrial fibrillation; PC-ASPECTS: Posterior circulation Alberta Stroke Program Early Computed Tomography Score; TIA: transient ischemic attack; OTA: estimated time of basilar artery occlusion to admission; NIHSS: National Institutes of Health Stroke Scale; OR: odd ratio; CI: confidence interval. Values were adjusted for age, sex, smoking, hypertension, hyperlipidemia, AF, baseline NIHSS score, intravenous thrombolysis, location of occlusion, OTA, history of stroke or TIA, baseline PC-ASPECTS, coronary heart disease.

**Fig. 2:**
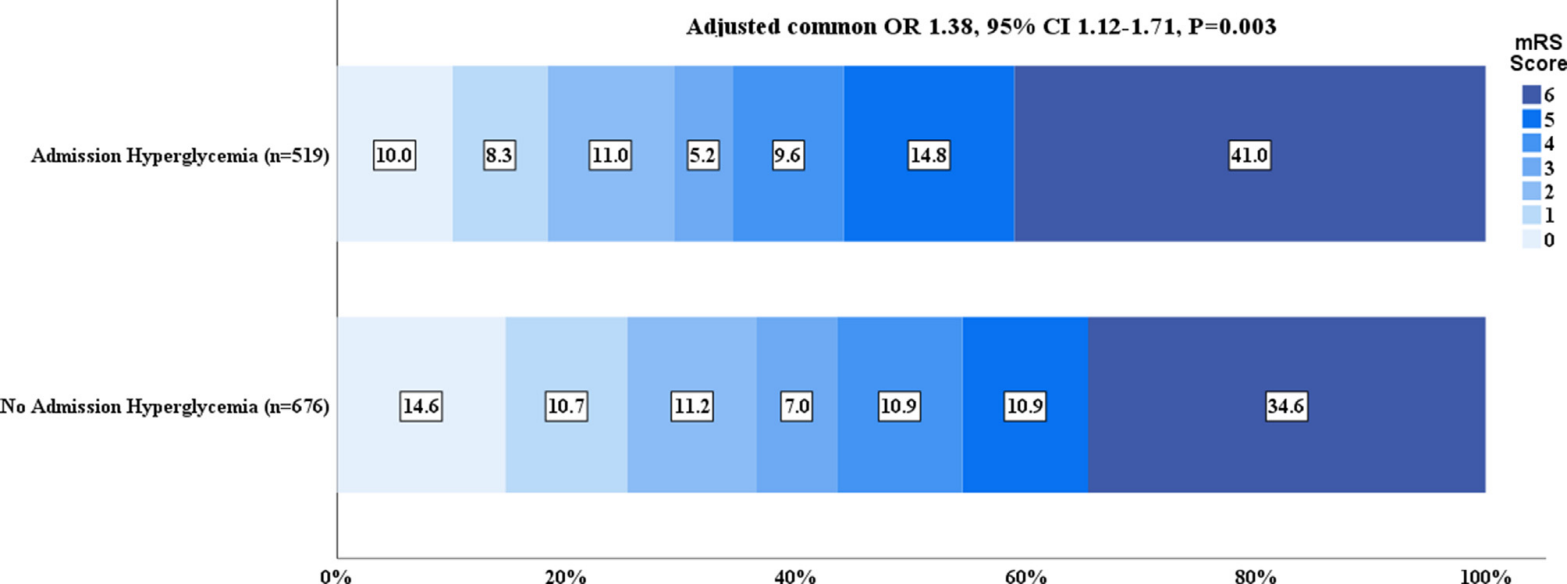
**Distribution of 90-day mRS scores according to hyperglycemia status in acute BAO patients treated with EVT**. The result of adjusted common odds ratio towards a worse outcome, with non-hyperglycemic patients as the reference group (adjusted common odds ratio 1.38; 95 ​% CI 1.12–1.71; P ​= ​0.003); mRS: modified Rankin Scale; BAO: basilar artery occlusion; EVT: endovascular treatment; CI: confidence interval.

In ordinal mRS shift analysis, after adjustment for confounding factors, hyperglycemia was associated with a shift towards worse functional outcomes at 90 days (adjusted common OR 1.38, 95 ​% CI 1.12–1.71, P ​= ​0.003; Fig. 2).

### Subgroup analysis and heterogeneous

**Fig. 3:**
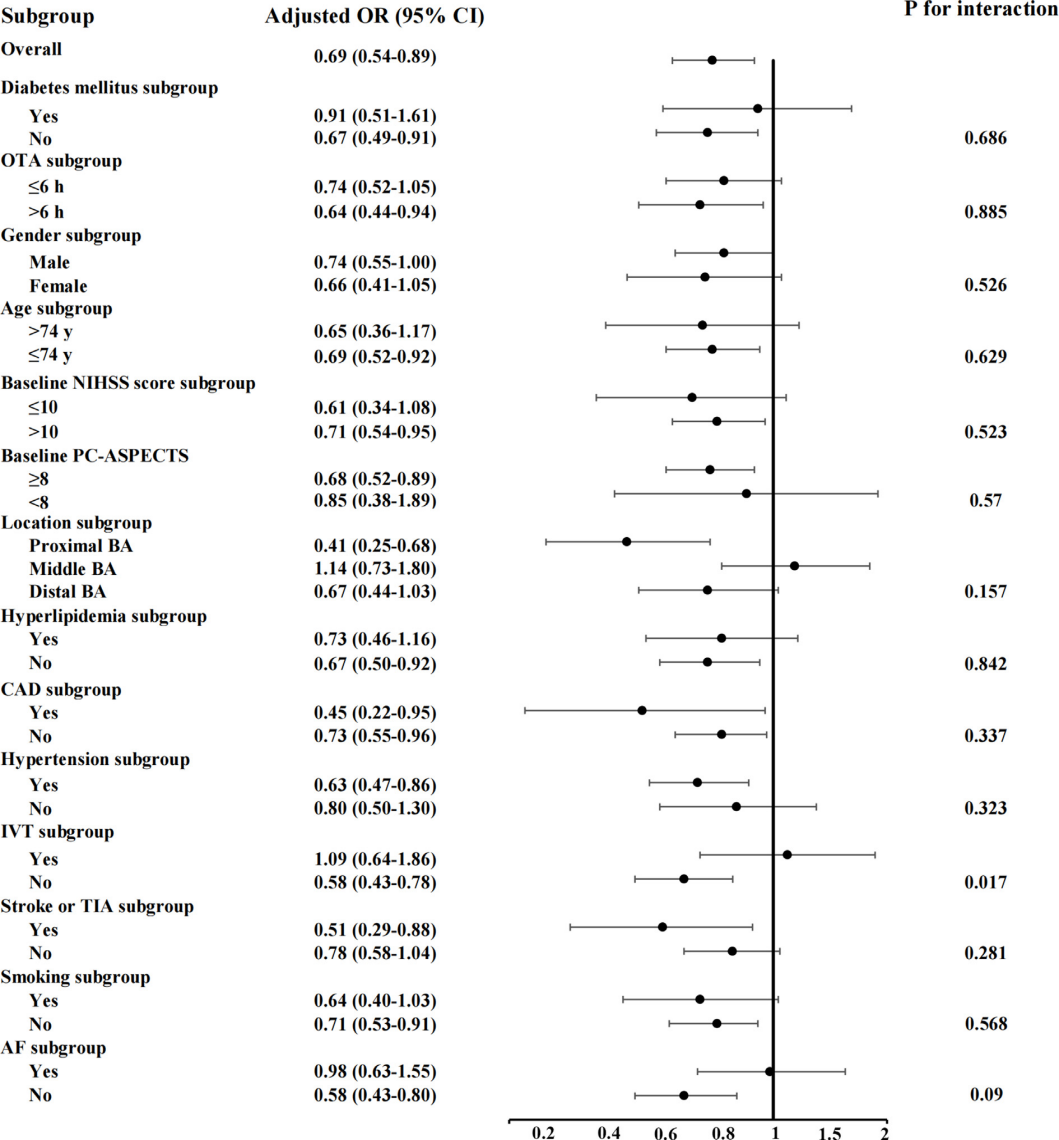
**Subgroup analysis for primary outcome (90-day modified Rankin Scale scores 0–3)**. This forest plot shows the differences in the primary outcome in all subgroups. OR: odd ratio; CI: confidence interval; OTA: estimated time of basilar artery occlusion to admission; NIHSS: National Institutes of Health Stroke Scale; PC-ASPECTS: Posterior circulation Alberta Stroke Program Early Computed Tomography Score; BA: basilar artery; CAD: coronary heart disease; IVT: intravenous thrombolysis; TIA: transient ischemic attack; AF: atrial fibrillation.

The relationship between hyperglycemia and primary outcome based on subgroup analysis was demonstrated in Fig. 3. We did an exploratory subgroup analysis stratified by age, gender, OTA, baseline NIHSS score, baseline PC-ASPECTS, level of occlusion sites, coronary heart disease, hyperlipidemia, hypertension, smoking, atrial fibrillation, IVT, DM, stroke or TIA. The multiplicative term was calculated for statistically heterogeneous. Significant interactions (statistically heterogeneous) were only observed in the IVT subgroup (P_interaction_ ​= ​0.017). We further analyzed this interaction effect in patients with different time windows (OTA ≤4.5 ​h and >4.5 ​h) (Supplementary Table 1). The interaction terms between hyperglycemia on admission and IVT allocation were significant for primary outcome in patients with OTA ≤4.5 ​h (P_interaction_ ​= ​0.019) but not in those with OTA >4.5 ​h (P_interaction_ ​= ​0.394).

Additionally, we further assessed the primary outcome of hyperglycemia on admission in patients with DM and those without DM. After adjusting for confounding factors, hyperglycemia on admission was associated with a decreased risk of favorable outcome in patients without DM, but not in those with DM (Without DM: aOR 0.67, 95 ​% CI 0.49–0.91, P ​= ​0.010; With DM: aOR 0.91, 95 ​% CI 0.51–1.61, P ​= ​0.737; Supplementary Table 2). Patients with DM were found to be inversely correlated with favorable outcome compared with those without DM (aOR 0.72, 95 ​% CI 0.54–0.97, P ​= ​0.030; Supplementary Table 2).

### Clinical outcomes for IVT and window time subgroups

Table 3 displays the association of hyperglycemia on admission and clinical outcomes in the subgroup of patients who underwent direct EVT vs. bridging therapy. Multi-variable logistic regression analysis confirmed that, for patients who received direct EVT, hyperglycemia on admission had a significant correlation with 90-day mRS scores 0–3, 0–1, and 0–2 (mRS score 0–3: aOR 0.58, 95 ​% CI 0.43–0.78, P ​< ​0.001; mRS score 0–1: aOR 0.53, 95 ​% CI 0.37–0.75, P ​< ​0.001; mRS score 0–2: aOR 0.60, 95 ​% CI 0.44–0.82; P ​= ​0.001). With respect to safety outcomes, after adjusting for confounding factors, hyperglycemia on admission was associated with an increased risk of 90-day mortality, but not sICH (aOR 1.42, 95 ​% CI 1.06–1.90, P ​= ​0.019). No differences were observed in the rate of successful recanalization between patients with and without hyperglycemia on admission. In addition, in patients who were treated with bridging therapy, there was no relationship of hyperglycemia on admission with the primary, secondary, or safety outcomes.

**Table 3:** Primary outcome, secondary outcomes, and safety outcomes for hyperglycemia in patients with acute BAO by direct EVT versus bridging therapy.

	Direct EVT				Bridging therapy (IVT ​+ ​EVT)			
	Crude OR (95 ​% CI)	P value	Adjusted OR (95 ​% CI)	P value	Crude OR (95 ​% CI)	P value	Adjusted Value (95 ​% CI)	P value
**Primary outcome**								
mRS 0–3 at 90 ​d	0.57 (0.43–0.76)	＜0.001	0.58 (0.43–0.78)	＜0.001	1.21 (0.76–1.93)	0.432	1.09 (0.64–1.86)	0.745
**Secondary clinical outcomes**								
mRS 0–1 at 90 ​d	0.52 (0.37–0.73)	＜0.001	0.53 (0.37–0.75)	＜0.001	1.32 (0.77–2.27)	0.315	1.20 (0.67–2.17)	0.539
mRS 0–2 at 90 ​d	0.59 (0.44–0.79)	＜0.001	0.60 (0.44–0.82)	0.001	1.30 (0.81–2.09)	0.278	1.22 (0.71–2.09)	0.470
Successful recanalization	0.84 (0.57–1.23)	0.370	0.89 (0.60–1.32)	0.555	0.85 (0.42–1.69)	0.634	0.91 (0.44–1.87)	0.793
**Safety outcomes**								
sICH at 3 ​d	1.08 (0.60–1.94)	0.802	1.02 (0.56–1.87)	0.939	1.63 (0.56–4.77)	0.373	1.58 (0.50–4.99)	0.432
Mortality at 90 ​d	1.46 (1.12–1.91)	0.006	1.42 (1.06–1.90)	0.019	0.83 (0.50–1.39)	0.479	0.78 (0.43–1.43)	0.425

BAO: basilar artery occlusion; OR: odd ratio; CI: confidence interval; EVT: endovascular treatment; IVT: intravenous thrombolysis; AF: atrial fibrillation; mRS: modified Rankin Scale; sICH: symptomatic intracranial hemorrhage, sICH: symptomatic intracranial hemorrhage; OTA: estimated time of basilar artery occlusion to admission; NIHSS: National Institutes of Health Stroke Scale; TIA: transient ischemic attack; PC-ASPECTS: Posterior circulation Alberta Stroke Program Early Computed Tomography Score. Values were adjusted for age, sex, smoking, hypertension, hyperlipidemia, AF, baseline NIHSS, location of occlusion, OTA, history of stroke or TIA, baseline PC-ASPECTS, and coronary heart disease.

Subgroups of different window times (OTA ≤4.5 ​h and >4.5 ​h) were also analyzed. In patients with OTA >4.5 ​h, hyperglycemia on admission had a significant correlation with 90-day mRS scores 0–3, 0–1, and 0–2 (mRS score 0–3: aOR 0.64, 95 ​% CI 0.46–0.90, P ​= ​0.010; mRS score 0–1: aOR 0.59, 95 ​% CI 0.39–0.88, P ​= ​0.009; mRS score 0–2: aOR 0.60, 95 ​% CI 0.42–0.86; P ​= ​0.005; Supplementary Table 3). No significant differences were observed in safety outcomes and successful recanalization. In patients with OTA ≤4.5 ​h, hyperglycemia on admission was not related to primary or secondary outcomes, or safety outcomes.

**Fig. 4:**
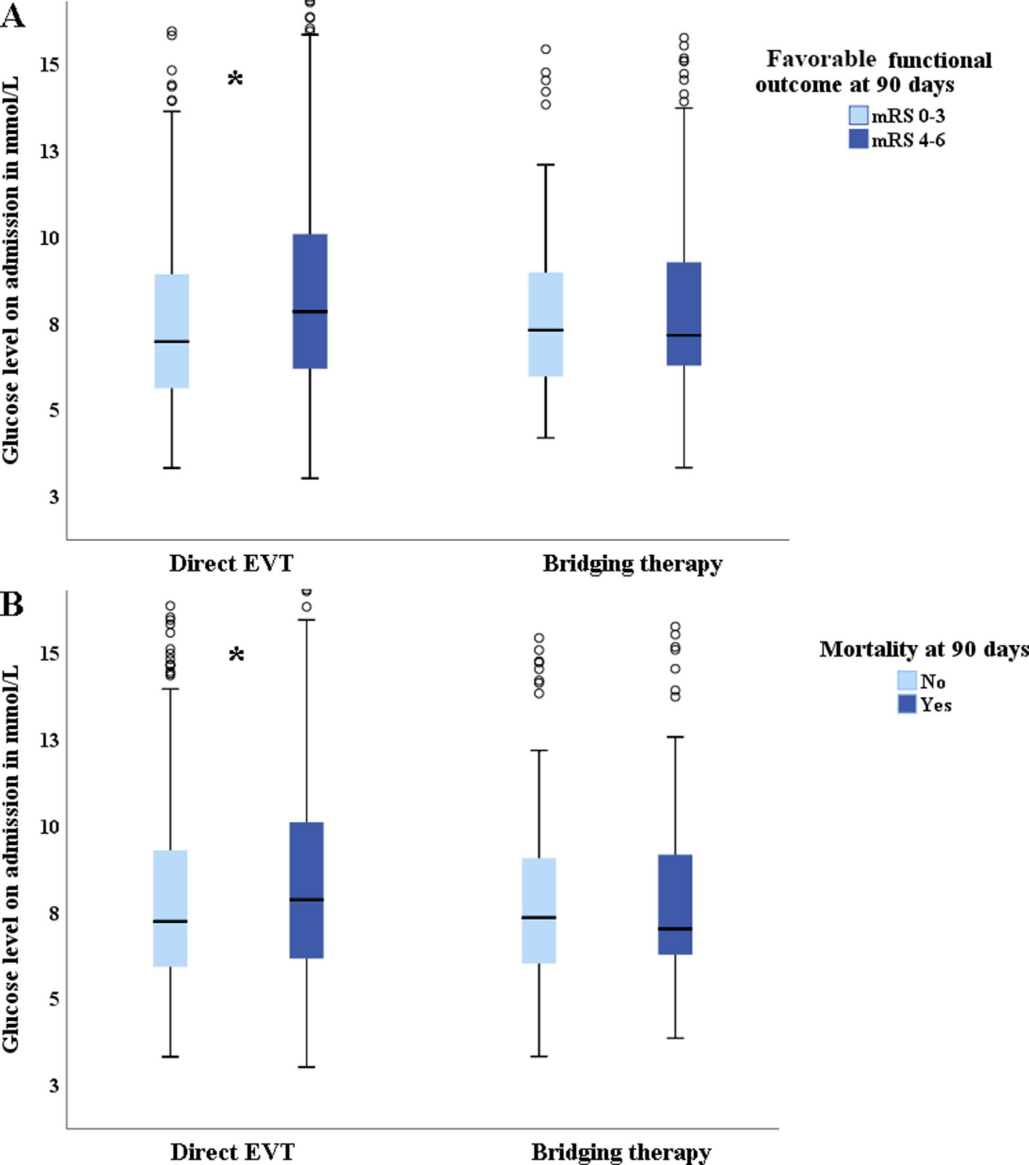
**Box plots of glucose concentration on admission for subgroups treated with direct EVT and bridging therapy showing favorable functional outcomes (A) and mortality (B) at 90 days in acute BAO patients.** ∗Significant difference at P ​< ​0.05; IVT: intravenous thrombolysis; EVT: endovascular treatment; mRS: modified Rankin Scale; BAO: basilar artery occlusion.

Box plots of the glucose level on admission in the subgroups treated with direct EVT versus bridging therapy, and their clinical outcomes are presented in Fig. 4. Patients with unfavorable functional outcomes (mRS score 4–6) had higher glucose levels on admission than those with favorable functional outcome (mRS score 0–3) in the group treated with direct EVT (P＜0.001). There was no difference in clinical outcomes for patients receiving bridging therapy (P ​= ​0.73). In addition, higher glucose levels on admission were associated with an increased 90-day mortality rate in patients receiving direct EVT (P ​= ​0.001), but not in those receiving bridging therapy (P ​= ​0.88).

### Association between admission glucose concentration and clinical outcomes

**Fig. 5:**
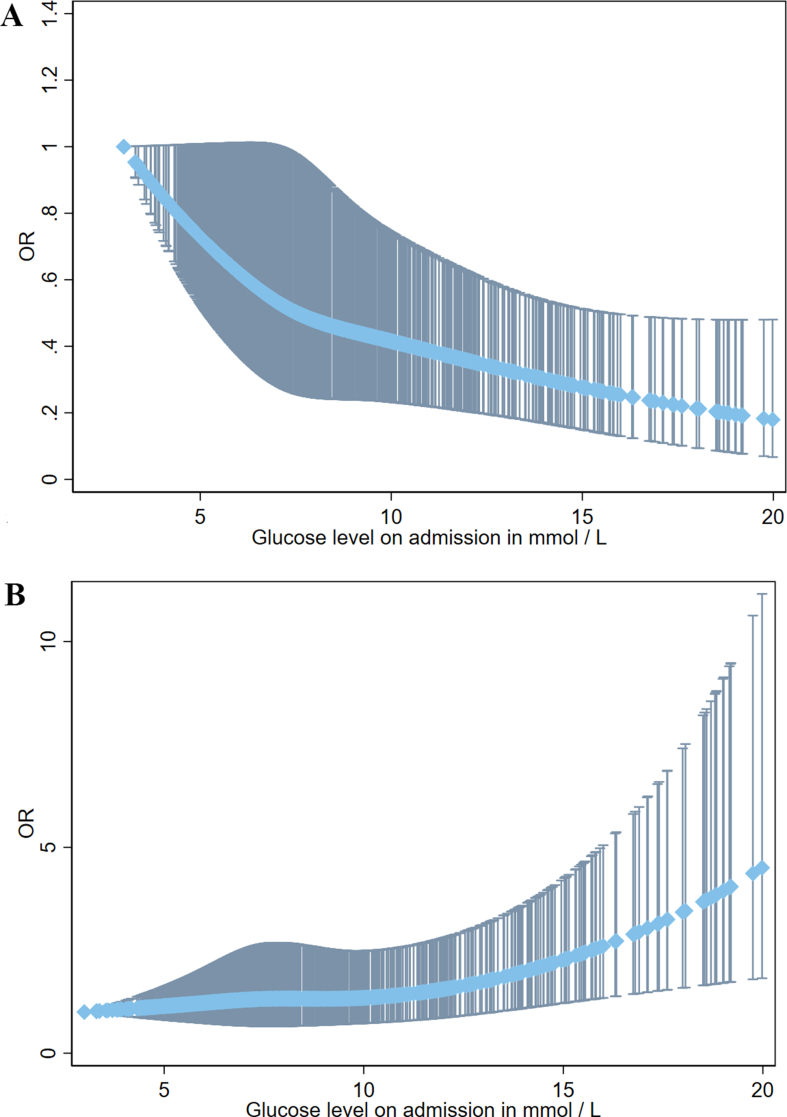
**The restricted cubic splines of favorable functional outcome (A) and mortality (B) at 90 days based on glucose levels on admission for acute BAO patients who underwent EVT.** The light blue line displays the adjusted OR and the dark blue line displays the 95 ​% confidence interval; OR: odd ratio; EVT: endovascular treatment; BAO: basilar artery occlusion.

The RCS of logistic regression model was used to evaluate the association of glucose levels on admission with favorable functional outcome and mortality at 90 days in patients receiving EVT (Fig. 5). After adjusting for confounding factors, there was a correlation between glucose levels on admission and lower ORs of favorable functional outcome at 90 days (aOR 0.91, 95 ​% CI 0.87–0.95, P＜0.001; per 1 ​mmol/L increase). A higher level of glucose on admission was correlated with an increased risk of mortality at 90 days (aOR 1.08, 95 ​% CI 1.03–1.12, P＜0.001; per 1 ​mmol/L increase). There was an incremental trend between odds of mortality at 90 days and admission glucose concentration. However, a downward trend was observed between 90-day favorable functional outcome and admission glucose concentration.

**Fig. 6:**
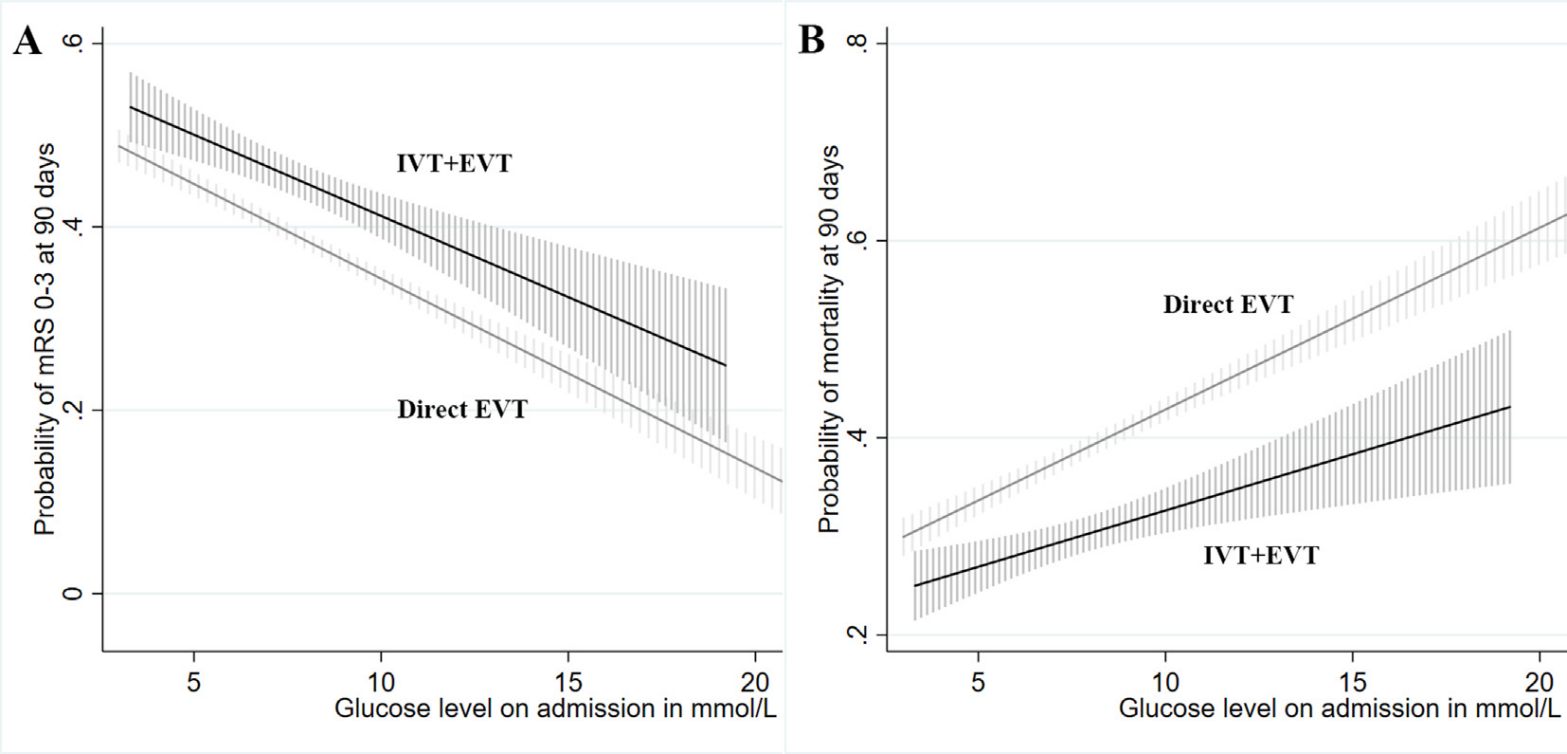
**Association of glucose levels on admission and IVT allocation with the probability of favorable functional outcome (A) and mortality (B) at 90 days**. Multivariable logistic regression plot to demonstrate the effect of glucose levels on admission (x axis) and IVT allocation on the probability of clinical outcomes. IVT ​+ ​EVT and direct EVT subgroups are indicated by the black line and the gray line, respectively. The dotted line displays the 95 ​% confidence interval. EVT: endovascular treatment; IVT: intravenous thrombolysis.

Furthermore, a multivariable logistic regression analysis was conducted to evaluate the effect of glucose levels on admission and IVT allocation on the likelihood of clinical outcomes (Fig. 6). Results show the incremental change of likelihood for 90-day mortality and based on glucose levels on admission separately for patients in patients treated with direct EVT and bridging therapy. These patients had a decreasing likelihood of 90-day favorable functional outcome and glucose levels on admission.

ROC analysis was used to evaluate the prognostic value of glucose levels on admission (AUC 0.58, 95 ​% CI 0.55–0.61). The optimal cut-off value for best discriminating 90-day unfavorable functional outcome was 7.395 ​mmol/L (Youden Index: 0.11; Sensitivity: 57 ​%; Specificity: 55 ​%).

## Discussion

Our nationwide prospective study, based on real-world data, indicated that hyperglycemia on admission was significantly correlated with worse functional outcomes at 90 days in patients with acute BAO treated with EVT. Furthermore, the effect of admission hyperglycemia appears to be modified by IVT allocation. In patients who received direct EVT, hyperglycemia on admission was associated with a higher proportion of 90-day worse functional outcomes and higher mortality than patients without admission hyperglycemia. There was no correlation between admission hyperglycemia and poor outcomes in patients treated with bridging therapy.

Accumulating studies have illustrated that hyperglycemia on admission was associated with worse functional outcomes among anterior circulation LVO patients both in East Asian and Caucasian populations [10,11,20,21]. The SWIFT (Solitaire Flow Restoration With the Intention for Thrombectomy) trial [21] and the MR CLEAN Registry (Multicenter Registry of Endovascular Treatment for Acute Ischemic Stroke in the Netherlands) [10] both reported that hyperglycemia on admission was significantly correlated to disability at 90 days in anterior circulation AIS patients who underwent EVT. Moreover, a meta-analysis showed that a high glucose level on admission was an independent predictor of unfavorable functional outcomes after EVT [13]. Yet no interaction between high glucose levels on admission and clinical or hemorrhagic outcomes was found in the MR CLEAN randomized trial cohort of patients with LVO undergoing EVT [22]. In line with the results of several reports on the anterior circulation, our study on patients with BAO receiving EVT supports the correlation between hyperglycemia on admission and worse functional outcomes.

The association of hyperglycemia on admission with safety outcomes after EVT for LVO remains controversial. The adverse effects of hyperglycemia on admission revealed by our study were limited to clinical rather than safety outcomes. Using data from the MR CLEAN registry, Rinkel LA et al. [10] found that hyperglycemia on admission was related to an increased risk of sICH and death in anterior circulation LVO patients treated with EVT. In addition, N Goyal et al. [12], in a study of 231 LVO patients treated with MT, demonstrated that hyperglycemia on admission was an independent predictor of 90-day mortality. A 10 ​mg/dL increase in admission blood glucose was associated with a higher likelihood of sICH. A similar relationship has also been described between baseline glucose and sICH in Chinese anterior circulation AIS patients treated with MT [23]. An observational study on patients with BAO treated with MT found that higher glucose levels on admission were related to a higher incidence of any ICH [24]. However, Kim et al. [21] did not find a correlation between baseline hyperglycemia with a higher mortality rate or sICH in the SWIFT trial. Accordingly, a meta-analysis did not demonstrate a difference in sICH rates between patients with DM or high admission glucose levels treated with MT [13]. Our findings [21] are in line with the results from the SWIFT study in anterior circulation LVO suggesting that hyperglycemia on admission was not associated with higher mortality or sICH in patients with acute BAO treated with EVT. The lack of an interaction of hyperglycemia and sICH in the posterior circulation (in contrast to that which had been observed with several reports in the anterior circulation) could be related to the fact that the incidence of ICH in BAO patients after EVT is lower than those in the anterior circulation [25]. This lower baseline incidence might reduce the statistical power to detect a significant relationship between hyperglycemia and sICH in acute BAO patients. In addition, we believe that methodological disparities and ethnic variations may also explain these results. These methodological disparities may be in terms of study design, patient selection criteria, outcome measurements, statistical analyses and adjustments for confounders. Ethnic variations in vascular anatomy, metabolic profiles, and genetic predisposition might modulate the impact of hyperglycemia on outcomes. Most hyperglycemia and sICH studies are based on Caucasians [10,12,21]. Different racial groups may also have distinct glucose metabolism profiles.

Significant interaction effect was found between the treatment effect of IVT and hyperglycemia on admission for predicting primary outcome. This interaction effect still remains within the 4.5-h window. This effect could have broader implications, as it occurs at the time which IVT is most likely to be effective. Our study could hint at a protective effect of IVT in the presence of hyperglycemia within the 4.5-h threshold, but this is speculative and requires more in-depth investigation. Additionally, we examined the subgroup of patients treated with direct EVT and bridging therapy. We observed a remarkable association of hyperglycemia on admission with low rates of favorable functional outcomes and high mortality rates in the direct EVT group, but not in the bridging therapy group. This differential might be explained by the fact that patients treated with alteplase presented in the earlier 4.5 ​h window compared to patients in the direct EVT group presenting in the 24 ​h window from estimated BAO symptom onset. As far as we are aware, there have been few studies on the possible modification of IVT effect by admission hyperglycemia. In stroke patients who are treated with IVT, hyperglycemia on admission has been recognized as an independent predictor of death, sICH, and poor functional outcomes [26]. However, the exact cause is still unknown and further investigation is needed.

Different window time subgroups were analyzed in our study. Admission hyperglycemia was correlated with functional outcomes in patients with OTA >4.5 ​h but not in those with OTA ≤4.5 ​h. Late window patients may be most affected by glucose due to its effects on infarct growth and collaterals [9,14]. Further studies may illuminate the underlying causes.

In accordance with prior studies [10,21,27], hyperglycemia on admission was not related to successful recanalization in our cohort. In contrast, a different study including 117 BAO patients (91 of which treated with MT) [14] demonstrated that lower baseline serum glucose levels were associated with successful vessel recanalization. In this study, Broocks et al. hypothesized that hyperglycemia may impede the fibrinolytic process, and thereby reduce the odds of reperfusion. Given the small sample size of BAO patients who had different treatments in this study, we believe their results should be interpreted with caution. In light of our study findings, it is likely that the correlation between admission hyperglycemia and poor clinical outcomes is not mediated by the lower likelihood of successful vessel recanalization.

The BASILAR (EVT for Acute Basilar Artery Occlusion Study) registry [28] suggested admission hyperglycemia increased the risk of poor functional outcomes and mortality in acute BAO patients undergoing EVT. Our results were inconsistent with the BASILAR registry. A sufficient sample size may be a reason for this, as our study has credible results. Moreover, the BASILAR registry identifies a critical risk factor for acute BAO. Our study moves a step forward by showing how that risk might be managed or manipulated by clinical interventions like IVT. The interaction between IVT and admission hyperglycemia suggests that clinicians need to consider the combined effects of blood glucose levels and thrombolysis treatment when planning a treatment strategy.

As a simple and readily available indicator, admission hyperglycemia provides a snapshot of the patient's status upon arrival. Moreover, admission glucose levels are routinely collected in most stroke centers. However, single isolated glucose reading can indeed be variable, comprehensive and continuous glycemic monitoring and management were really important in clinical practice. Hemoglobin A1c provides an excellent reflection of long-term glucose control. Periodic glucose readings could provide more comprehensive data during the hospital stay. All of those indicators can provide a more rounded view of the glycemic status, but not represent the precise glycemic status at the onset of BAO. The lack of hemoglobin A1c and periodic glucose readings makes it difficult to evaluate clinical outcomes via those valuable glycemic monitoring.

We recognize several limitations in our study. First, the observational design may lead to selection bias. Second, the measurement of glucose levels on admission were measured under different conditions. Baseline serum glucose levels may be influenced by food intake, dining time, admission time, and glucose lowering medications. Third, East Asian populations have a higher proportion of intracranial atherosclerotic disease than Western populations. Our results may not be applicable to other ethnic groups. Fourth, some confounding variables such as pre-stroke mRS were not included in our study, which could influence the effects of hyperglycemia seen in EVT patients with BAO. Fifth, a low IVT rate and insufficient statistical power in a small subgroup may limit conclusions from generalization. Finally, information about continuous glucose monitoring, hypoglycemic drug therapy and hemoglobin A1c levels was not recorded. It is unknown whether an acute initial elevation in glucose or persistent rise in glucose over time plays a role in patient outcomes. Including these important variables in future studies could confirm our results and examine whether treatment with glycemic lowering medication may further modify outcomes.

In conclusion, in patients with acute BAO undergoing EVT in the ATTENTION registry, hyperglycemia on admission was independently correlated with worse functional outcomes at 90 days, more so in patients who underwent direct EVT. There was no association of baseline hyperglycemia on recanalization or sICH. The effect of admission hyperglycemia appears to be modified by IVT allocation.

## Funding

This study was funded by National Natural Science Foundation of China (No. 82001452 and 81900530), Fundamental Research Funds for the Central Universities (No. YD9110002014) and Natural Science Foundation of Anhui Province (No. 2108085MH272).

## Declaration of competing interest

The authors declare no competing interests.
