# Supplementary material for: Effects of admission hyperglycemia and intravenous thrombolysis allocation in acute basilar artery occlusion after endovascular treatment: Analysis of the ATTENTION registry
Source: Neurotherapeutics. 2023 Dec 19;21(1):e00303. doi: 10.1016/j.neurot.2023.10.013 (PMC10903088; doi:10.1016/j.neurot.2023.10.013)
Supplement: Multimedia component 1 [file mmc1.doc]

**Supplementary Materials**

**Supplementary Table 1**. The primary outcome of hyperglycemia on admission stratified by IVT allocation subgroups by OTA ≤4.5 hours and >4.5 hours

|  | Crude OR (95% CI) | P value | Adjusted OR (95% CI) | P value | P for interaction |
| --- | --- | --- | --- | --- | --- |
| **OTA ≤4.5 hours** |  |  |  |  |  |
| With IVT | 1.21 (0.72-2.03) | 0.477 | 1.08 (0.59-1.95) | 0.807 | 0.019 |
| Without IVT | 0.41 (0.25-0.70) | 0.001 | 0.49 (0.27-0.91) | 0.024 |
|  |  |  |  |  |  |
| **OTA >4.5 hours** |  |  |  |  |  |
| With IVT | 1.30 (0.42-4.01) | 0.649 | 1.01 (0.19-5.24) | 0.993 | 0.394 |
| Without IVT | 0.65 (0.47-0.91) | 0.010 | 0.63 (0.44-0.89) | 0.009 |

IVT: intravenous thrombolysis; OTA: estimated time of basilar artery occlusion to admission; OR: odd ratio; CI: [confidence interval](javascript:;); NIHSS: National Institutes of Health Stroke Scale; PC-ASPECTS: Posterior circulation Alberta Stroke Program Early Computed Tomography Score. Values were adjusted for age, sex, smoking, hypertension, hyperlipidemia, atrial fibrillation, baseline NIHSS, location of occlusion, history of stroke or transient ischemic attack, baseline PC-ASPECTS, and coronary heart disease.

**Supplementary Table 2**. The primary outcome of hyperglycemia on admission in patients with DM and without DM

|  | Crude OR (95% CI) | P value | Adjusted OR (95% CI) | P value |
| --- | --- | --- | --- | --- |
| **Overall** |  |  |  |  |
| Patients with DM vs. Patients without DM | 0.81 (0.61-1.06) | 0.119 | 0.72 (0.54-0.97) | 0.030 |
|  |  |  |  |  |
| **Patients with DM** |  |  |  |  |
| Admission  hyperglycemia vs. No admission hyperglycemia | 0.87 (0.51-1.46) | 0.590 | 0.91 (0.51-1.61) | 0.737 |
|  |  |  |  |  |
| **Patients without DM** |  |  |  |  |
| Admission  hyperglycemia vs. No admission  hyperglycemia | 0.66 (0.49-0.88) | 0.004 | 0.67 (0.49-0.91) | 0.010 |

DM: diabetes mellitus; OR: odd ratio; CI: [confidence interval](javascript:;); OTA: estimated time of basilar artery occlusion to admission; NIHSS: National Institutes of Health Stroke Scale; PC-ASPECTS: Posterior circulation Alberta Stroke Program Early Computed Tomography Score. Values were adjusted for age, sex, smoking, hypertension, hyperlipidemia, atrial fibrillation, baseline NIHSS score, intravenous thrombolysis, location of occlusion, OTA, history of stroke or transient ischemic attack, baseline PC-ASPECTS, coronary heart disease.

**Supplementary Table 3**. Primary outcome, secondary outcomes, and safety outcomes for hyperglycemia in acute BAO patients with EVT subgroups by OTA ≤4.5 hours and >4.5 hours

| OTA ≤4.5h | | | | | OTA >4.5h | | | |
| --- | --- | --- | --- | --- | --- | --- | --- | --- |
|  | Crude OR (95% CI) | P value | Adjusted OR (95% CI) | P value | Crude OR (95% CI) | P value | Adjusted Value (95% CI) | P value |
| **Primary outcome** |  |  |  |  |  |  |  |  |
| mRS 0-3 at 90 d | 0.70 (0.48-1.00) | 0.051 | 0.78 (0.52-1.16) | 0.221 | 0.68 (0.50-0.93) | 0.014 | 0.64 (0.46-0.90) | 0.010 |
| **Secondary clinical outcomes** |  |  |  |  |  |  |  |  |
| mRS 0-1 at 90 d | 0.74 (0.48-1.13) | 0.158 | 0.88 (0.55-1.40) | 0.582 | 0.61 (0.42-0.89) | 0.011 | 0.59 (0.39-0.88) | 0.009 |
| mRS 0-2 at 90 d | 0.85 (0.58-1.23) | 0.375 | 1.01 (0.66-1.53) | 0.972 | 0.64 (0.46-0.89) | 0.008 | 0.60 (0.42-0.86) | 0.005 |
| Successful recanalization | 0.83 (0.49-1.41) | 0.496 | 0.88 (0.51-1.54) | 0.665 | 0.85 (0.55-1.30) | 0.448 | 0.92 (0.59-1.43) | 0.717 |
| **Safety outcomes** |  |  |  |  |  |  |  |  |
| sICH at 3 d | 1.65 (0.75-3.64) | 0.215 | 1.69 (0.74-3.85) | 0.214 | 0.94 (0.47-1.86) | 0.848 | 0.82 (0.41-1.66) | 0.588 |
| Mortality at 90 d | 1.29 (0.90-1.86) | 0.162 | 1.08 (0.72-1.63) | 0.699 | 1.34 (0.98-1.83) | 0.068 | 1.36 (0.97-1.92) | 0.075 |

BAO: basilar artery occlusion; EVT: endovascular treatment; OTA: estimated time of basilar artery occlusion to admission; OR: odd ratio; CI: [confidence interval](javascript:;); mRS: modified Rankin Scale; sICH: symptomatic intracranial hemorrhage; NIHSS: National Institutes of Health Stroke Scale; PC-ASPECTS: Posterior circulation Alberta Stroke Program Early Computed Tomography Score. Values were adjusted for age, sex, smoking, hypertension, hyperlipidemia, atrial fibrillation, baseline NIHSS, intravenous thrombolysis, location of occlusion, history of stroke or transient ischemic attack, baseline PC-ASPECTS, and coronary heart disease.
